# Clinical Society of Maryland

**Published:** 1892-03

**Authors:** W. T. Watson

**Affiliations:** Secretary; 1603 N. Broadway, Baltimore


### CLINICAL SOCIETY OF MARYLAND.

The Two Hundred and Sixtieth Regular Meeting, held in Baltimore January 15, 1892. Vice-President R. B. Norment, in the Chair.

Dr. L. McLane Tiffany read a paper entitled "Skin Dimpling in Carcinoma of the Female Mamma."

Dr. Geo. J. Preston read a paper on "Hemorrhage into the Substance of the Spinal Cord, with Report of a Probable Case."

Dr. Harry Friedenwald reported several cases of atropine intoxication from the use of eye-drops.

Dr. R. M. Hall related a case of periodic insanity, associated with salpingitis of the right side, and asked about the advisability of removing the uterine appendage for relief of the mental trouble. Patient has had three attacks, one of which is now on her. They occur about the 11th or 12th of the month. Menstruation occurs at the first of the month. All that now remains of the salpingitis is an enlargement in the right side.

Dr. W. S. Gardner: Although the attacks may not come on at the time of menstruation, yet the two things may be related. If these attacks come on every four weeks, and the woman has menstrual pains regularly, there is a relation of time, if nothing else, and it is possible that by careful watching a connection could be established between them.

Dr J. F. Martinet: All physicians, and especially gynecologists, recognize that a condition may be developed about the uterus and its appendages which can excite acute mania. I do not arise to answer Dr. Hall's question, but to relate a case in my own practice. I attended the woman in confinement. Her husband confided to me that she was a very willful woman, and had had two attacks of acute mania, each associated with childbirth. During this confinement, no symptoms of mental trouble appeared. On recovery, she went on a visit to the South, her husband remaining at home. On her return some months later, she was in a nervous condition, and finding some things at home not to her liking, she became morose. I saw her on Friday. By Sunday she was in a state of acute mania. Dr. Rohe, of the State Insane Asylum, believes the mental trouble to be due to uterine excitement, and suggests the removal of the ovaries. I am inclined to think it would be better to postpone the operation for a time. The woman should bear no more children.

Dr. J. E. Michael: I think it is admitted by those who have given the subject attention that the insanity in women due to the puerperal state, pregnancy and diseases of the genital apparatus is a condition which develops in persons predisposed to insanity by inheritance. A case came under my care last summer of a very intelligent young lady who was married under rather unfavorable circumstances, a good deal of excitement attending the wedding. The groom took his bride to his home in Virginia. In one week from the time of the marriage, he brought her back perfectly insane. Her family had heard of the removal of ovaries for the cure of insanity, and suggested this treatment. I opposed it, because I did not think it an operation which ought to be considered under the circumstances. Previous insanity in the family was denied, but, upon careful investigation, I found that this young lady had suffered previous from insanity, lasting several months; also that an uncle had been insane for a number of years; her father, also, had several attacks of insanity. I regarded it as hereditary insanity, the exciting cause being the excitement attending the marriage. I suggested an asylum, and was dismissed. Doctor Wilson got the case, and I was able to follow its course. In three months the symptoms gradually cleared away. I think there is great probability that insanity will again develop.

In Dr. Hall's case there was salpingitis, in which the acute symptoms have passed away, and there is some residuum of the inflammation. The woman has become insane. Her uncle has been insane. She evidently has a predisposition to insanity, and this particular condition has been the exciting cause. I am very much inclined to the view that if this woman had been subjected to ophorectomy, or any other excitement, that insanity would have been the result.

I do not think the operation is indicated in this case more than in the case of any other exciting cause. I think the operation is decidedly contra-indicated.

The data given by Dr. Hall do not seem to show a definite relation to the menstrual flow. The case is to be regarded simply as one of insanity.

Dr. J. H. Branham: Dr. Michael, judging from the two cases related, seems to be opposed to operation for mental trouble. There is no question but that there are some cases in which benefit is derived from such operation. On the other hand, there are doubtless a great many cases that would be made worse. I know of a case in which mental trouble developed in a young woman which was thought to be associated with some abnormal condition of the uterine appendages. Their removal was effected and the woman developed violent mania just after the operation, and she died in this condition.

If cases are clearly associated with abnormal conditions about the uterus, especially attacks of melancholia occurring near the menstrual period, it seems, from the report of many cases, that great benefit is sometimes derived from these operations.

As to the case in point, there is not enough information before the society to decide whether there is any relation between the menstrual flow and attacks of insanity. Cases of acute salpingitis tend to recover very often if let alone, but frequently the apparent recovery is only a period of relief, and without any other infection from some exciting cause, such as overwork, the trouble recurs and operation may have to be done. In Dr. Hall's case, I think it would be better to wait and see if the mental symptoms cannot be cured without operation. Certainly an operation should not be done within three months.

Dr. A. J. Preston: I think the point that Dr. Michael partly brought out, viz: the careful consideration of the personal and family history of the patient, is most important. There may have been certain things in the life of the individual which may have caused insanity, such as a period of trouble, emotional excitement, great poverty, or some other decided shock. Where the family history shows distinct heredity, I perfectly agree with Dr. Michael that there should, as a rule, be no attempt to cure by operation. The relationship between the higher brain centers and the genital organs is very close, and irritation of one may cause disturbance in the other. Some of these cases, pointing to uterine irritation, amount simply to hysterical mania. I have seen two or three cases that were relieved temporarily by hypnotism. I saw one in Charcot's clinic suffering with violent mania who was perfectly rational the next day after being hypnotized. Since then I have run across cases now and then in which the hysterical symptoms amounted almost to mania; these symptoms would subside at certain times and were closely related to some genital irritation.

Where there is no distinct hereditary predisposition, and where there is in the patient's personal history the evidences of decided genital irritation, and there is a chance of this irritation still continuing, it would seem to me that an operation would promise the best results. Cases of recurrent mania are among the most obstinate of all forms of mental diseases. If an operation would hold out any hope I should be in favor of advising it, and particularly early in the case. An operation later may do no good, just as in epilepsy an early operation may do good, while a later one may not. As there is in the case under discussion some genital irritation persisting, and the attacks of insanity are periodical, I should be strongly in favor of operation.

Dr. Michael, replying to the remarks of Dr. Branham, said: I did not mean to go into the discussion of operating in cases where there is distinctly a relation between menstruation and the mental condition. I did not deem it a part of the present discussion. I should not hesitate to perform laparotomy in cases where these things exist.

Dr. W. G. Townsend related a case which was under his observation for about three months. A young lady, aged about 23, had hysterical attacks periodically and finally was the subject of acute mania. She had marked tenderness in the right ovarian region. She has been placed in an asylum and her ovaries removed. The results of the operation were not yet known to Dr. Townsend, but would be ascertained and reported to Dr. Hall.

Dr. Hall: The tumor in the lady's side does not seem to trouble her at all.

I would like to ask if there are any cases on record where perfect recovery has ensued after operation?

Dr. Preston: There are a certain number of such cases on record in which operations have cured the mental symptoms. The operation has not been as successful as was hoped for, perhaps, because it has been performed rather indiscriminately; but there are undoubted cases which have resulted very favorably.

W. T. WATSON, M. D.,
1603 N. Broadway, Baltimore. Secretary.

---

